# Differences in neuropsychological and behavioral parameters and brain structure in patients with familial adenomatous polyposis: a sibling-paired study

**DOI:** 10.1186/s13053-016-0060-7

**Published:** 2016-10-10

**Authors:** Ana Sánchez Azofra, Trilokesh D. Kidambi, Rita J. Jeremy, Peggy Conrad, Amie Blanco, Megan Myers, James Barkovich, Jonathan P. Terdiman

**Affiliations:** 1 Cruces University Hospital, Medical Faculty, Basque Country University, Biscay, Spain; 2 Division of Gastroenterology, University of California, San Francisco, 1701 Divisadero, San Francisco, CA 94115 USA; 3 Pediatric Clinical Research Centery, Clinical and Translational Science Institute, University of California, San Francisco, San Francisco, CA USA; 4 Hereditary GI Cancer Prevention Program, Helen Diller Family Comprehensive Cancer Center, University of California, San Francisco, San Francisco, CA USA; 5 Division of Pediatric Neuroradiology, University of California, San Francisco, San Francisco, CA USA

**Keywords:** Familial adenomatous polyposis (FAP), Adenomatous polyposis coli (APC), Hereditary colon cancer, Neuropsychology, Emotional functioning

## Abstract

**Background:**

Familial adenomatous polyposis (FAP) is an autosomal dominant hereditary colon cancer syndrome caused by mutations in adenomatous polyposis coli (APC) with both colonic and extra-colonic manifestations. Case reports have noted an association with FAP and intellectual disability and animal studies have shown that APC is implicated in neural development and function, but no studies have investigated neuropsychological, behavioral, or structural brain characteristics of patients with FAP.

**Methods:**

We undertook a pilot, sibling-pair study comparing three patients with FAP to their sex-matched siblings without FAP. Each sibling pair underwent neuropsychological testing by a blinded examiner, high resolution brain MRI scans, and the mother of each pair rated her children’s adaptive life skills and behavioral and emotional characteristics. Given the small number of study participants in this pilot study, quantitative comparisons of results were made by subtracting the score of the non-FAP sibling from the FAP patient on the various neuropsychological tests and parent rating questionnaires to calculate a difference, which was then divided by the standard deviation for each individual test to determine the difference, corrected for the standard deviation. Diffusion numbers in multiple regions of the brain as assessed by MRI were calculated for each study participant.

**Results:**

We found similarity between siblings in all three pairs on a wide range of neuropsychological measures (general intelligence, executive function, and basic academic skills) as tested by the psychologist as well as in descriptions of adaptive life skills as rated by mothers. However, mothers’ ratings of behavioral and emotional characteristics of two of the three pairs showed differences between the siblings, specifically that the patients with FAP were found to have more behavioral and emotional problems compared to their siblings. No differences in brain structure were identified by MRI.

**Conclusion:**

We report the first study exploring neuropsychological, behavioral, emotional, and structural brain characteristics of patients with FAP and found subjective differences as assessed by maternal perception in behavioral and emotional characteristics in patients with FAP compared to their siblings. Larger studies are needed to elucidate the relationship, if any, between FAP and brain function.

## Background

Familial adenomatous polyposis (FAP) is an autosomal dominant hereditary colon cancer syndrome characterized by 100 or more premalignant polyps caused by germline mutations in the tumor suppressor gene, adenomatous polyposis coli (APC), located on chromosome 5q21-q22 [1, 2]. While there is near-complete penetrance of the colonic manifestations, there is variable penetrance of the extra-colonic manifestations of the disease and the location of the mutation within the APC gene is associated with the severity of colonic polyposis, the degree of cancer risk, the age of cancer onset, survival, and the presence and frequency of extra-colonic manifestations [3].

Case reports have linked FAP to the presence of intellectual disability (formerly called mental retardation) [1, 4–10]. In each of these cases, the individuals had a deletion of all or a portion of chromosome 5q, but the nature of the intellectual disability was poorly defined and it remained unclear whether the intellectual disability was secondary to loss of APC gene function or another genetic defect due to the chromosomal loss. APC is known to be involved in regulating a variety of cellular processes, including mitosis, cytoskeletal dynamics, axonogenesis, cell polarity and apoptosis [11–14] and is central to the WNT signaling pathway, mediating the destruction of cytoplasmic β-catenin protein [15]. The APC protein has also been found to be an essential regulator, in vivo, of synaptic density, maturation and signal transduction networks in forebrain neurons [16]. Furthermore, studies in mice with a mutated APC gene have shown learning and memory impairments, autistic-like behaviors, increased repetitive behaviors, reduced social interest, increased locomotor activity as well as abnormal brain morphology and function [16, 17].

In our work caring for multiple families with FAP, we have made the informal observation that FAP-affected individuals appear to have more cognitive and social-emotional difficulties than their non-affected relatives, but this clinical observation has not been verified with formal testing. Determining whether cognitive or behavioral problems are part of the phenotypic spectrum of FAP clearly has important clinical implications and early recognition of these issues would allow for interventions to potentially mitigate the problems. We, therefore, undertook a pilot sibling-pair study with the aim of assessing feasibility of identifying differences in neuropsychological performance, social-emotional characteristics, and of brain morphology assessed with high resolution MRI, of individuals with and without FAP. To our knowledge, this is the first such study.

## Methods

### Study participants

Three FAP families were selected to participate in the study, based on convenience. In each family, two young adult siblings of the same sex, who were within seven years of age of one another, one with confirmed FAP and the other with negative germline testing for FAP, comprised the sibling pair. Each sibling pair shared the same biological parents and grew up in the same household. The siblings underwent a comprehensive battery of neuropsychological testing as well as high resolution MRI of the brain. The mother of each sibling pair completed a set of questionnaires describing her children’s adaptive life skills and social- emotional characteristics. All study participants underwent informed consent and this study was approved by the Institutional Review Board of the University of California, San Francisco (UCSF).

### MRI Technique

All MRI scans were performed on a GE 3T MR scanner (General Electric Healthcare) with an eight-channel phase array head coil and included volumetric T1 images (inversion recovery prepared fast spoiled gradient-recalled echo, TR = 11.58 ms, TE = 4.8 ms, inversion time = 450 ms, partition size = 0.895 mm, in-plane resolution = 0.41 mm], T2 images (volumetric fast spin-echo, TR = 4.0 s, TE = 104 ms, contiguous 1.5-mm sections, in-plane resolution =0.94 mm]. High-angular resolution diffusion MRI (HARDI) data were acquired for DTI analyses (b = 2000 s/mm2, 55 directions, TR/TE = 15,000/74 ms, 2-mm isotropic voxels). HARDI data were processed with a weighted least-squares fit to compute the diffusion tensor metrics. ROIs were drawn on the locations that correspond to anterior corona radiata and posterior corona radiata and in the larger surrounding left and right anterior frontal white matter and left and right posterior parieto-occipital white matter regions, the genu and splenium of the corpus callosum, and bilateral cingulum.

#### Neuropsychological tests and parent rating questionnaires

Baseline demographic data and medical and family history were obtained on all of the participants. A psychologist (RJJ) blinded to the study participants’ medical history examined all sibling pairs and administered tests of general intelligence (Wechsler Abbreviated Scale of Intelligence-Second Edition - WASI-II) [18], executive function (Delis-Kaplan Executive Function System - D-KEFS) [19], and basic academic skills (Wechsler Individual Achievement Test-Third Edition - WIAT-III) [20]. The mothers of the sibling pairs rated their children on questionnaires of adaptive life skills (Vineland Adaptive Behavior Scales, Second Edition - Vineland-II-Parent/Caregiver Rating Form) [21] and social-emotional characteristics (Vineland-II and ASEBA Adult Behavior Checklist for Ages 18–59 - ABCL/18-59) [22].

### Data analysis

All of the tests administered yielded standardized age-normalized scores, though each test had its own mean and standard deviation (SD) - for example, for the WASI-II the mean was 100 and SD was 15 and for the D-KEFS the mean was 10 and SD was 3. Furthermore, a higher score indicated better functioning on some measures but poorer on others. Consequently, the absolute scores were not directly comparable across the measures. Given the small number of study participants in this pilot study and the many measures, we did not perform statistical analyses of the differences between the siblings. Instead, we focused on the pattern of the quantitative within sibling-pair differences in scores on the many measures by constructing a common metric: units of standard deviation of within-sibling pair difference. Specifically, for each pair of siblings we calculated the difference between their standardized scores on the given measure and divided the difference by the standard deviation of that measure. For example, on the VCI (Verbal Comprehension Index) of the WASI-II test that had a standard deviation of 15 and a higher score was consistent with better function, the patient with FAP of family 1 scored 76 while the sibling without FAP scored 81, corresponding to a within-sibling pair difference of −0.33 SD. A standard deviation score less than zero indicated that the sibling with FAP showed poorer functioning than the sibling without FAP. Diffusion numbers (average diffusivity, fractional anisotropy, radial and axial diffusivity) in multiple regions of the brain as assessed by MRI were calculated for each study participant.

## Results

### Clinical and genetic profiles

#### Family 1

**Fig. 1:**
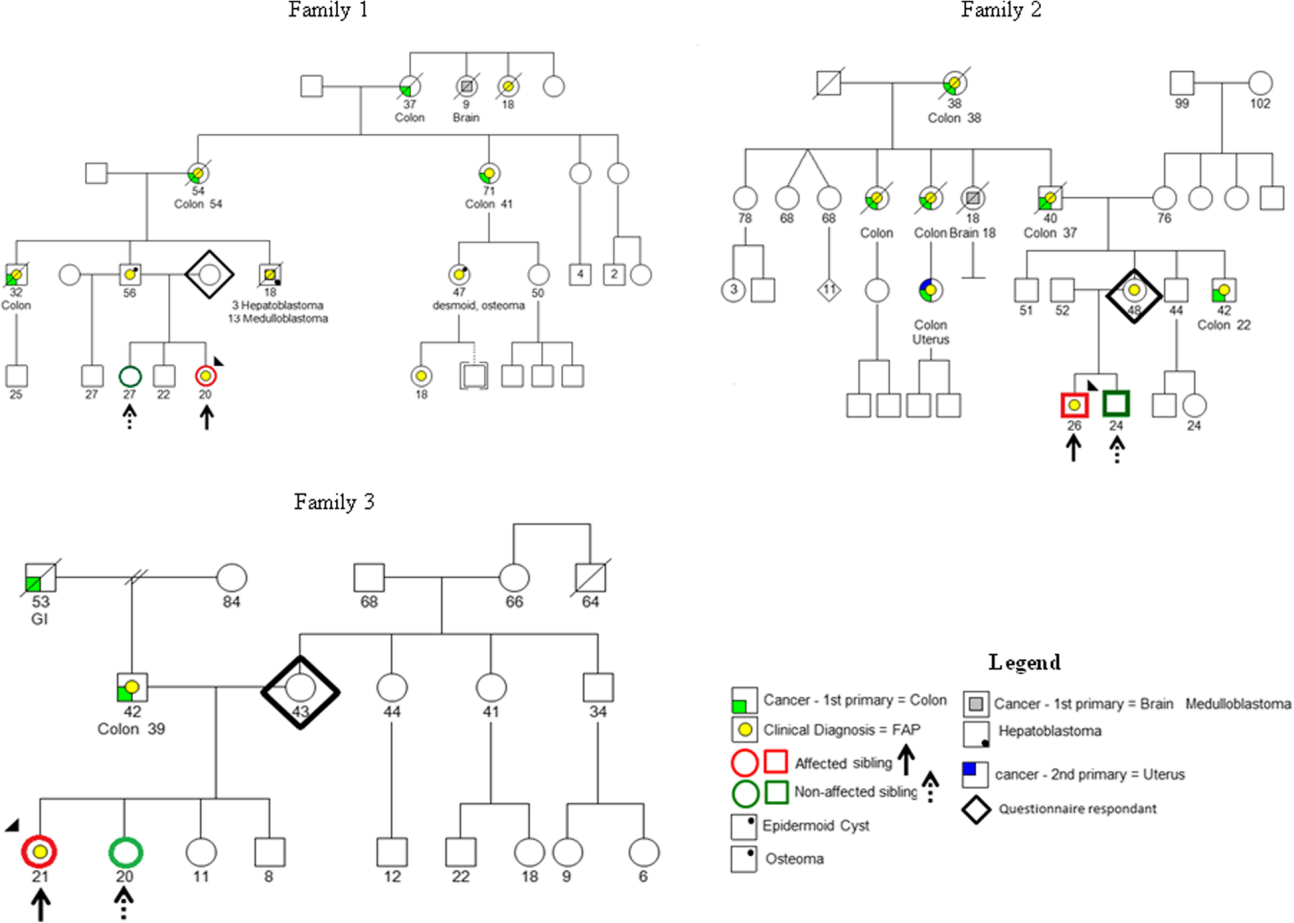
Pedigree for each family

The proband was a 20-year-old Caucasian woman who underwent genetic testing at the age of seven due to a family history of FAP and tested positive for the known family APC Q1045X mutation. Her first colonoscopy was performed at the age of ten and multiple sessile adenomas were found throughout the colon. The patient is screened annually with upper endoscopy and colonoscopy and has not yet had prophylactic colectomy. Her paired sibling was her 27-year-old sister, who had negative genetic testing for the family mutation at age ten. A pedigree is shown in Fig. 1.

#### Family 2

The proband was a 26-year-old man with mixed Hispanic, European and Native American ancestry who underwent genetic testing for FAP at the age of seven due to a family history of FAP and tested positive for the known APC N1124X mutation. This patient had a total restorative proctocolectomy with ileoanal J-pouch at the age of eight. His paired sibling was his 24-year-old brother, who had negative genetic testing for the family mutation at the age of nine. A pedigree is shown in Fig. 1.

#### Family 3

The proband was a 21-year-old woman of mixed Asian, European, and Hispanic ancestry who was diagnosed with FAP at the age of 16 after endoscopy two years earlier for hematochezia, anemia and weight loss revealed greater than 100 gastric polyps and greater than 200 colonic polyps. Genetic testing for FAP was positive for the APC S457X mutation. She underwent total colectomy with ileal pouch-anal anastomosis at the age of 19. Her father was subsequently diagnosed with FAP and he tested positive for the known family APC S457X mutation. Her paired sibling was her 20-year-old sister, who had negative genetic testing for the family mutation at age 18. A pedigree is shown in Fig. 1.

#### Results of neuropsychological testing and of parental ratings of adaptive, behavioral, and emotional characteristics

**Fig. 2:**
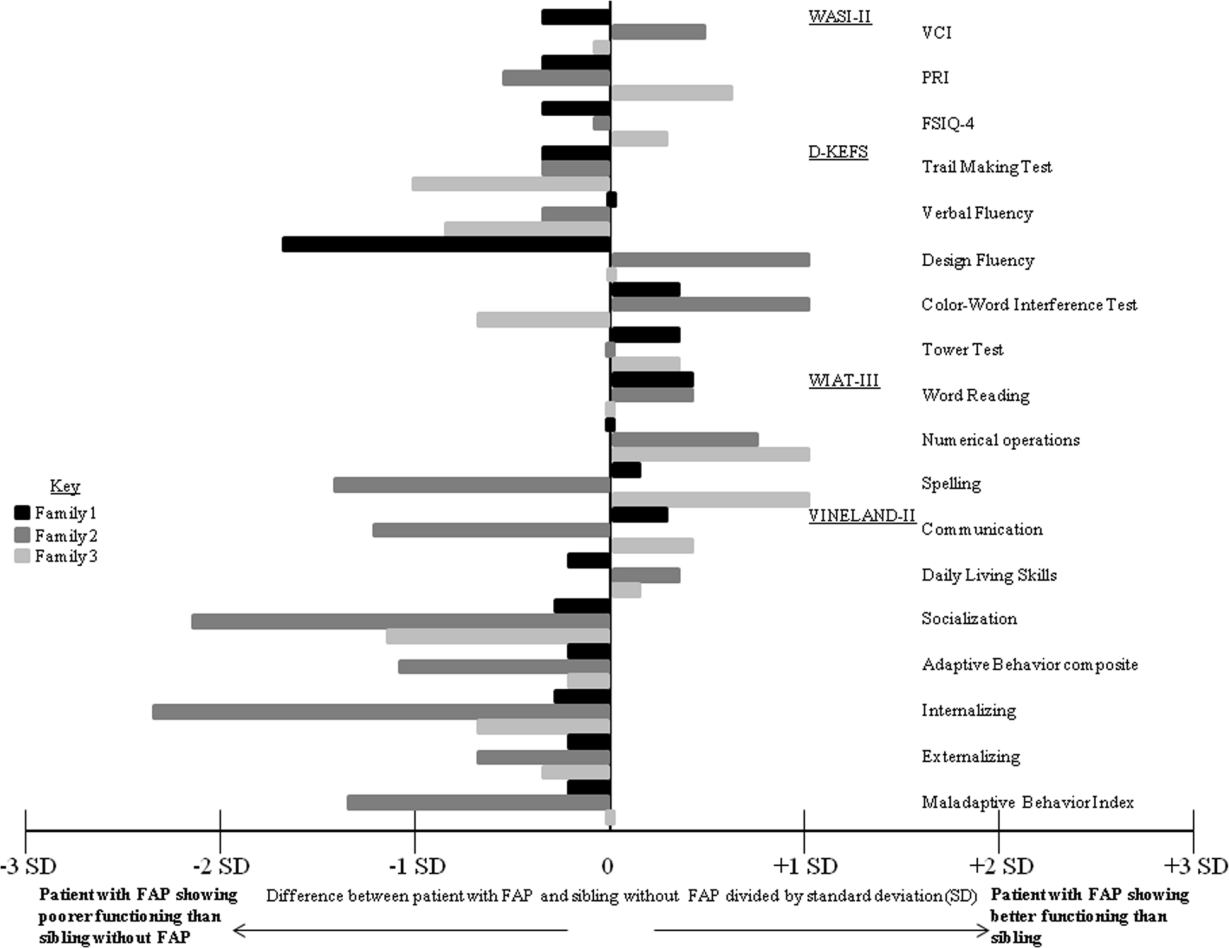
Differences in results of neuropsychological and adaptive life skills testing. VCI-Verbal Comprehension Index; PRI-Perceptual Reasoning Index; FSIQ4-Full Scale Intelligence Quotient of all 4 subtests

**Fig. 3:**
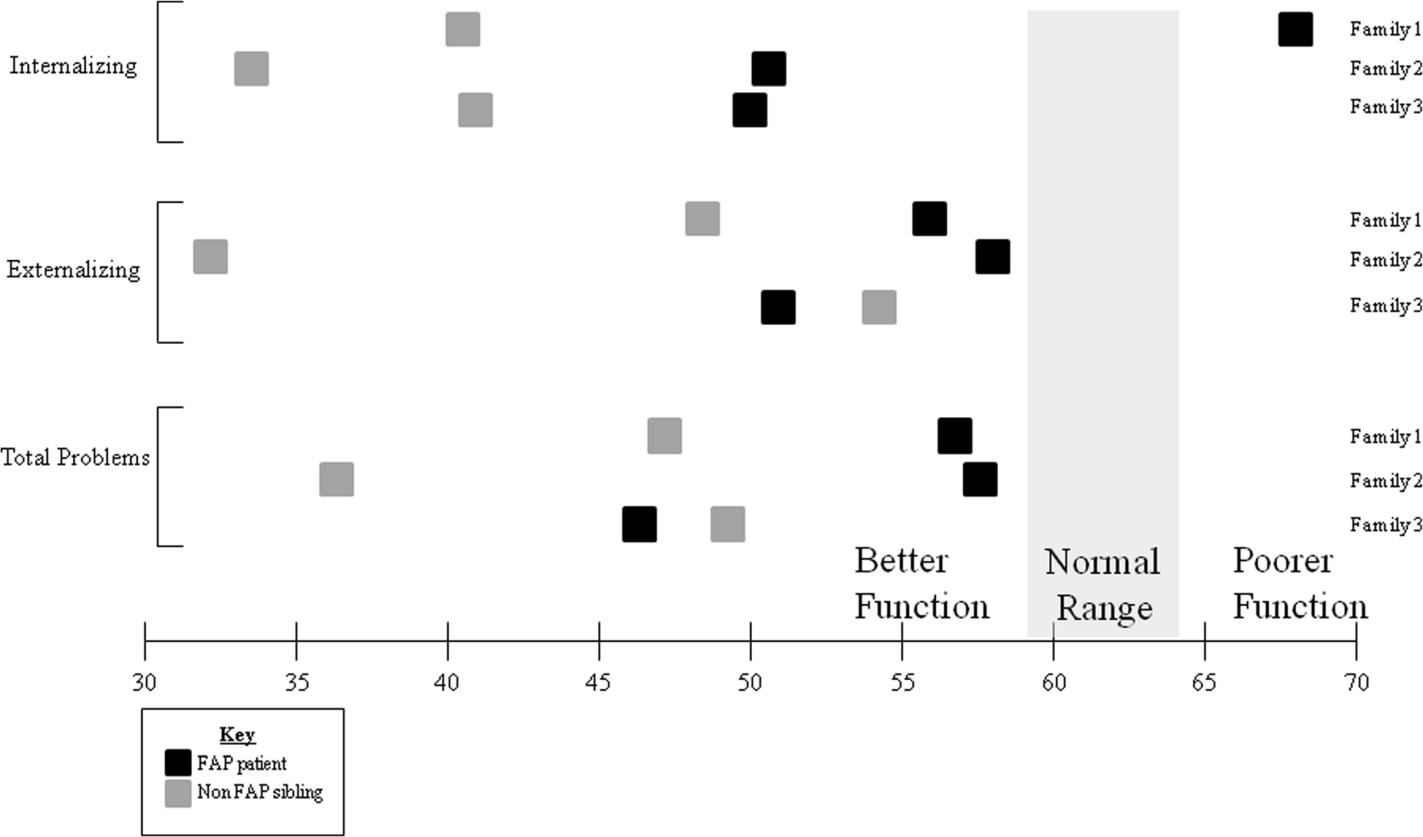
Results of mothers’ ratings of behavioral and emotional characteristics (ABCL/18-59) of patients with FAP and siblings without FAP. Normal range 59–64. Scores higher than 69 are suggestive of poorer function and scores lower than 59 are suggestive of better function

The results of neuropsychological testing of the sibling pairs are shown in Fig. 2. The sibling pairs scored similarly on the WASI-II (general intelligence), D-KEFS (executive function), and WIAT-III (academic skills) tests, with only a single notable difference in family 2, where the patient with FAP scored five points lower than his sibling pair on the design fluency portion of the D-KEFS test, corresponding to a difference of 1.67 standard deviations. In contrast, the mothers’ rating of their children did show differences. The results of questions related to adaptive life skills measured by the Vineland-II are shown in Fig. 2. Overall, there was a trend towards the mothers’ rating their child with FAP with poorer scores compared to their child without FAP. This was most noticeable in family 2, where the mother noted poorer functioning of her son with FAP compared to her son without FAP (as evidenced by a difference in greater than one standard deviation) in five of the eight domains measured. Figure 3 shows the results of mothers’ ratings of behavior and social-emotional characteristics as measured by the ABCL/18-59. In Families 1 and 2, there was a consistent trend towards the mothers’ rating their child with FAP with poorer function than their child without FAP on all aspects tested.

#### Results of MRI

The diffusion numbers in multiple regions of the brains showed no difference in the white matter metrics in all three pairs between the FAP patients and non-FAP siblings. No differences were detected using diffusion tensor imaging analysis.

## Discussion

We report the first study exploring neuropsychological, social-emotional, and structural brain characteristics of patients with FAP. Based on informal clinical observation, we expected within-pair differences favoring improved function in the non-FAP sibling across many domains. However, we found differences in only a subset of the measured parameters. Compared to sex-matched siblings of similar age with negative germline mutations for FAP, two of the three patients with FAP were rated by their mothers as showing more problematic behavioral and social-emotional characteristics. In contrast, there were similarities on the many measures of neuropsychological functioning (general intelligence, executive function, and basic academic skills) tested by the blinded psychologist and on ratings of skills of daily life by the mothers. These results do not fully support our hypothesis that patients with FAP would show poorer functioning compared to their unaffected siblings.

There is a biological basis for the role of APC in neurocognitive development. APC expression by adult neural stem cells is a critical component of adult neurogenesis in mice through regulation of intracellular β -catenin levels [13] and lack of APC induces severe laminary defects in some regions of the mouse brain, including the cerebral cortex, hippocampus, olfactory bulbs and cerebellum [11]. Additionally, APC is required for multiple aspects of early cerebral cortical development in mice, including the regulation of cell number, interkinetic nuclear migration, cell polarity and cell type specification [12]. Furthermore, APC mutant mice have abnormal brain function and behavior including depression-like behavior and decreased social interaction [16] as well as autistic-like behaviors as manifested by increased repetitive behaviors and reduced social interest [17]. Studies in humans have thus far been limited to case reports, including a patient with FAP secondary to deletion of the entire long arm of chromosome 5 leading to a phenotype of FAP with features of Prader-Wili Syndrome [4] and another patient with deletion of the entire APC gene through loss of 5q21-22 with the classic FAP phenotype along with intellectual disability and dysmorphic features [5]. In contrast to the animal studies that have shown abnormal neurocognitive development in APC-mutant mice and limited case reports in humans, the results of this study show no systematic deficits in patients with FAP compared to their non-FAP siblings in performance on neuropsychological tests, suggesting a possible compensatory genetic mechanism in humans that leads to a normal neurocognitive phenotype.

There were many strengths to the study design that deserve mention. First, the paired-sibling design was an efficient and natural study design that allowed for control over the environment in which the paired subjects were raised and therefore accounted at least partially for this potential confounder. Second, multiple aspects of neurocognitive functioning were assessed with validated and accepted neuropsychological tests addressing multiple domains; widely-used and accepted rating questionnaires allowed the mothers to describe their children’s typical behavior in natural settings of home and school, with family and friends, and MRI was utilized to assess brain structure. Third, a single psychologist administered all of the neuropsychological tests to all the patients and their siblings and she was blinded to their medical status (FAP or non-FAP), which also allowed for mitigation of potential observer bias. There was potential for bias to have accounted for the differences identified between sibling pairs by the mothers’ rating, given the mother had knowledge of whether her children had FAP. In two of these families, the children were diagnosed at a young age and this may have not only affected the mother’s perception of her child but may have affected the treatment of the child growing up as a result of the diagnosis which may have affected the patient’s behavior and social skills.

The major limitation of the study was the small sample size, which could not provide sufficient power to detect a difference. The aim was to carry out a pilot study and we found that the study was feasible. It is possible that differences in cognitive phenotype do exist but were not assessed in the validated measures s utilized in this study. Given the well-accepted and widely available measures and methods used in this study, this template could be adopted in a larger study, which would have greater power to detect any significant differences.

## Conclusions

Our pilot study did not detect differences on a variety of neurocognitive skills or in brain structure as assessed by MRI in patients with FAP compared to their unaffected siblings. However, a pattern of poorer functioning of the FAP patients in social and emotional characteristics as rated by their mothers was identified. The study was underpowered to detect significant differences. Further research with a larger sample of matched pairs is needed to elucidate the relationship, if any, between FAP and the development of the brain and on cognitive skills and on social-emotional behaviors. Our preliminary data suggest that it may be worthwhile to focus on measures of social-emotional functioning in planning future studies.

## Abbreviations

ABCL/18-59: Adult Behavior Checklist for Ages 18–59
APC: Adenomatous polyposis coli
D-KEFS: Delis-Kaplan Executive Function System
FAP: Familial adenomatous polyposis
HARDI: High-angular resolution diffusion
SD: Standard deviation
VCI: Verbal comprehension index
Vineland-II: Vineland Adaptive Behavior Scales, Second Edition
WASI-II: Wechsler Abbreviated Scale of Intelligence-Second Edition
WIAT-III: Wechsler Individual Achievement Test-Third Edition
